# The genetics of colony form and function in Caribbean *Acropora* corals

**DOI:** 10.1186/1471-2164-15-1133

**Published:** 2014-12-17

**Authors:** Elizabeth M Hemond, Stefan T Kaluziak, Steven V Vollmer

**Affiliations:** Marine Science Center, Northeastern University, Nahant, MA USA

**Keywords:** Coral Reefs, *Acropora*, Cnidaria, Gene expression, RNA-seq

## Abstract

**Background:**

Colonial reef-building corals have evolved a broad spectrum of colony morphologies based on coordinated asexual reproduction of polyps on a secreted calcium carbonate skeleton. Though cnidarians have been shown to possess and use similar developmental genes to bilaterians during larval development and polyp formation, little is known about genetic regulation of colony morphology in hard corals. We used RNA-seq to evaluate transcriptomic differences between functionally distinct regions of the coral (apical branch tips and branch bases) in two species of Caribbean *Acropora*, the staghorn coral, *A. cervicornis*, and the elkhorn coral, *A. palmata*.

**Results:**

Transcriptome-wide gene profiles differed significantly between different parts of the coral colony as well as between species. Genes showing differential expression between branch tips and bases were involved in developmental signaling pathways, such as Wnt, Notch, and BMP, as well as pH regulation, ion transport, extracellular matrix production and other processes. Differences both within colonies and between species identify a relatively small number of genes that may contribute to the distinct “staghorn” versus “elkhorn” morphologies of these two sister species.

**Conclusions:**

The large number of differentially expressed genes supports a strong division of labor between coral branch tips and branch bases. Genes involved in growth of mature *Acropora* colonies include the classical signaling pathways associated with development of cnidarian larvae and polyps as well as morphological determination in higher metazoans.

**Electronic supplementary material:**

The online version of this article (doi:10.1186/1471-2164-15-1133) contains supplementary material, which is available to authorized users.

## Background

Colonial modular organisms, such as corals, bryozoans, and tunicates, are formed from groups of asexually produced, genetically identical modules (‘polyps’ or ‘zooids’) that are interconnected to produce an integrated super-organism [1]. Modules are connected by living tissue, which allows intra-colony communication, resource sharing [2], and in some cases a coordinated division of labor (DOL) permits specific modules to specialize in feeding, reproduction or defense [3, 4]. The coordination of growth among modules can create diverse colony morphologies, such as those in tropical reef-building corals ranging from simple hemispherical and plating colonies to complex branching colonies.

Scleractinian (hard) coral colonies have a simple body plan of polyps and connecting tissue overlaid on top of a secreted aragonite (calcium carbonate) skeleton. The coral animal is comprised of anemone-like polyps with a gastro-vascular cavity formed by two cell layers (ectoderm and endoderm) separated by a layer of mesogloea. The polyps are interconnected by a layer of tissue (coenosarc) overlaying the skeleton, but may also share coelenteric canals within the skeleton [5]. Colony integration and inter-polyp communication is evident in the elaborate colony morphologies formed by corals, which often serve as key identifying characteristics of species [6]. However, within species some morphological variation may also occur due to environmentally-induced phenotypic plasticity [7, 8] or genetic polymorphism.

The genetic mechanisms underlying colony growth are poorly understood, but cnidarian genomes are known to contain many of the key genes expressed during coordinated development of bilaterians, such as Hox/ParaHox [9], Hedgehog [10], Wnt [11–13], TGFß/BMP [14, 15], Notch [16, 17] and other developmental signaling pathways [18–22]. Some of these developmental genes, including Hox/ParaHox and Wnt genes, have been shown to function in axial patterning of the solitary freshwater hydrozoan *Hydra* [23–26], the colonial marine hydrozoan *Hydractinia echinata* [27, 28], the solitary marine anthozoan *Nematostella vectensis* [12, 29], as well as in the early developmental stages of scleractinian corals [30, 31]. A ParaHox gene, *cnox-2*, has also been associated with DOL among zooids in hydrozoans [32, 33], likely relating to the development or exclusion of oral structures. Additionally, characteristic gene expression differences have been observed between swimming (nectophore) and feeding (gastrozooid) zooids in siphonophores (Hydrozoa) [4]. These findings suggest that other colonial cnidarians, including some corals, may show polyp-specific transcription. The genetics of coral development has been examined through post-larval settlement [34, 35], yet little is known about the basis of either colony coordination or DOL in mature colonies.

Branching *Acropora* corals are a good system for studying genetic regulation of growth form and colony coordination in anthozoans, because corals in this genus (with the exception of the subgenus *Isopora*) exhibit dimorphic polyp types. *Acropora* corals have axial polyps with six tentacles located at the apical tip of their branches and radial polyps with twelve tentacles located along the sides of the branches. Axial polyps are typically the site of rapid growth and have lighter coloration due to a lower concentration of symbiont algae (i.e. *Symbiodinium* or zooxanthellae) [2, 36–40]. In contrast, radial polyps are generally smaller and have darker pigmentation corresponding to higher symbiont densities. Actively growing branch tips in *Acropora* corals are usually sterile, and gamete production occurs only in mature radial polyps [36, 41–43]. *Acropora* have diversified into the most specious genus of scleractinian corals with over 120 described species and growth forms ranging from arborescent, such as *A. cervicornis*, to more tabulate forms, such as *A. palmata*. The success of these growth forms has enabled *Acropora* corals to colonize a wide range of habitats and become dominant reef-building corals both in the Indo-Pacific and the Caribbean. However, the genetic basis of this DOL within the colony remains unknown. One microarray study examined differential gene expression within *A. millepora* and found few differences between bases and tips of the branches. Differences were limited to lysosome lipase activity and fluorescence [44], which do not explain the large functional differences within the colony.

**Figure 1:**
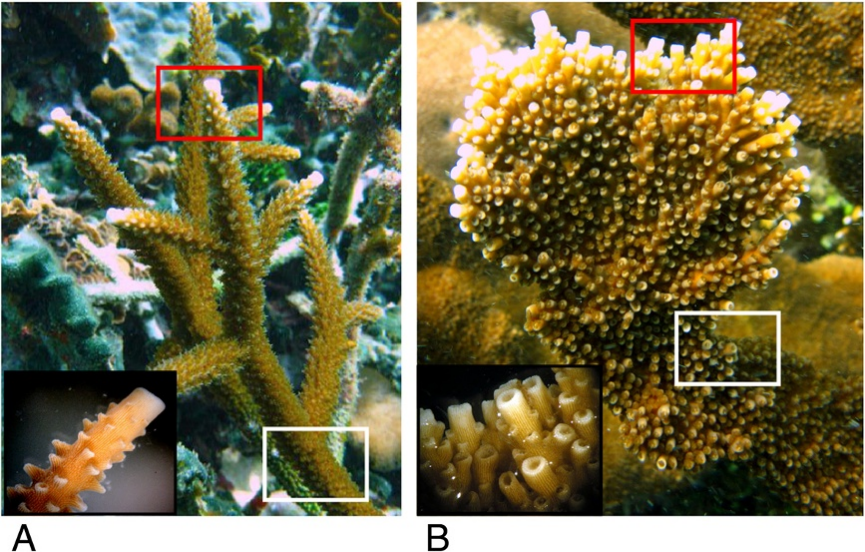
**Branch morphology of** ***A. cervicornis*** **(A) and** ***A. palmata*** **(B), with insets showing branch tips.** Regions of sampling for branch tips (red boxes) and bases (white boxes).

Despite the extensive species diversity globally, the Caribbean has only two *Acropora* species (Figure 1), the staghorn coral, *A. cervicornis,* and the elkhorn coral, *A. palmata*, which are known to hybridize, generating an intermediate morphology hybrid called *A. prolifera* [45]. These two sister species, which have distinct morphologies allowing them to occupy different habitats, are thought to have diverged over three million years ago, when *A. palmata* first appears in the fossil record [46]. *A. palmata,* which has robust branches with fused axial polyps, inhabits the shallow, high-energy reef crest, whereas *A. cervicornis,* which has thin branches generally dominated by a single axial polyp, tends to inhabit the fore-reef and back-reef habitats.

High-throughput sequencing facilitates the characterization and quantification of whole transcriptomes in non-model organisms and is a powerful new tool for studying species of conservation concern, such as *Acropora* corals. This technology is already being used to study issues such as coral response to climate change [47–49] and disease [50], and is the best currently available method to comprehensively study total transcriptomic variation. In this study, we used Illumina RNA sequencing (RNA-seq) to examine within-colony gene expression differences in the two Caribbean *Acropora* species, comparing actively growing apical branch tips to less actively growing branch bases. We examined overall gene expression differences within the coral colonies and between species, and we evaluated the differentially expressed (DE) genes for patterns relating to growth, polyp development, and deposition of a mineralized skeleton. Here we describe and focus on (1) genes that are DE between branch bases and branch tips for both species (i.e. DE by colony position), which indicate processes that contribute to or arise from DOL within the colony, and (2) genes that are DE by both colony position and species, which indicate processes that may be involved in facilitating morphological differences between species.

## Results and discussion

### Transcriptome-wide RNA-seq profiles

The RNA-seq libraries contained an average of 4.7 million reads, with 3.6 million mapped reads (see Additional file 1). The combined *A. cervicornis* and *A. palmata* coral-only dataset included 47,748 transcripts, of which 23,554 transcripts were expressed at least 100 normalized counts, and 22,320 transcripts remained after removing those with group SD > mean. Of the 22,320 transcripts in the coral dataset, nearly 15,000 were annotated with known or predicted proteins at an e-value < 10^−5^ in the UniProt database (see Additional file 2).

**Figure 2:**
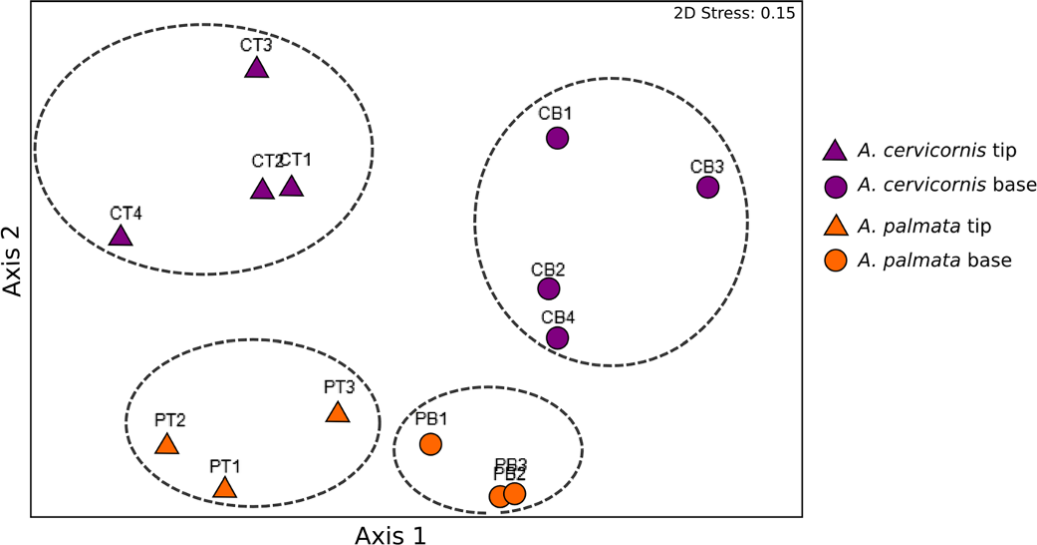
**nMDS for all samples and transcripts expressed at > 100 total normalized counts (n = 23,554).** Dashed lines delineate groups of samples.

Non-metric multidimensional scaling (nMDS) analyses of the gene expression profiles show that the samples form distinct groups by species (*A. cervicornis* and *A. palmata*) and by colony position (tip and base) (Figure 2). PERMANOVA analyses indicate that polyps sampled from different locations along the colony branch have highly different gene expression profiles (d.f. = 1, Pseudo-F = 4.4169, P = 0.001), as do polyps sampled from the two species (d.f. = 1, Pseudo-F = 5.8765, P = 0.001). However, there was no significant interaction between these factors for the transcription profiles as a whole (d.f. = 1, Pseudo-F = 0.9161, P = 0.480).

### Differentially expressed genes

**Figure 3:**
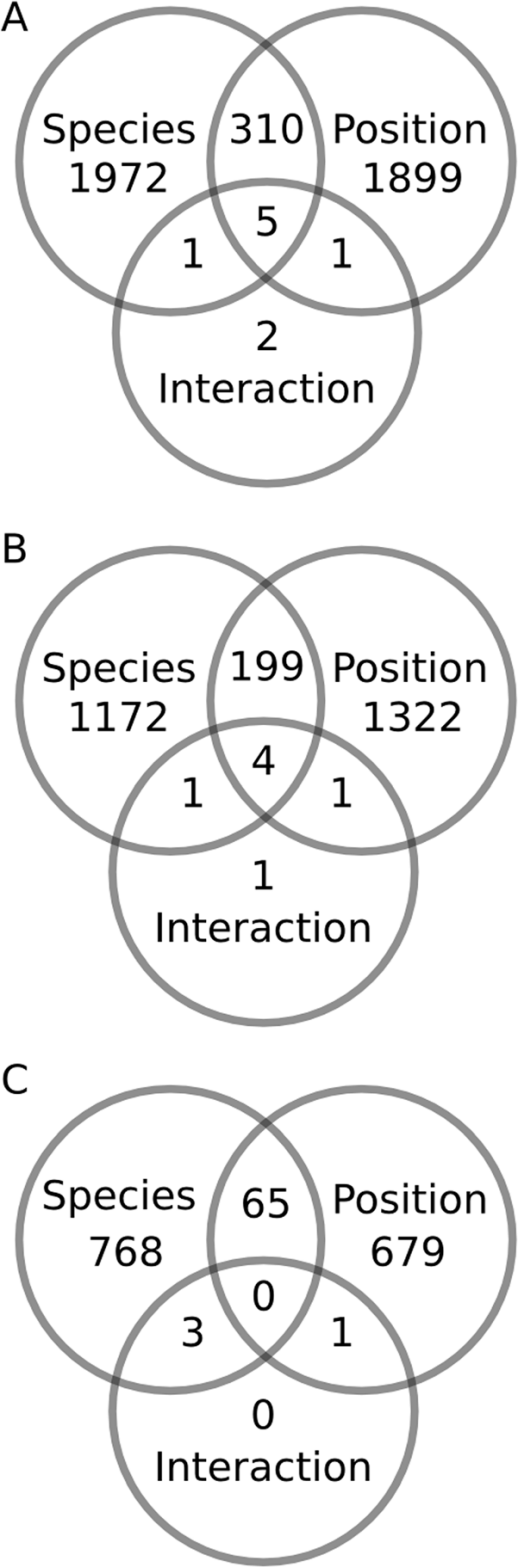
**Number of DE genes for factors (colony position and species) and their interaction.** Venn diagrams include results for all coral transcripts **(A)**, annotated transcripts **(B)**, and annotated transcripts with greater than 2-fold difference between treatments **(C)**.

**Figure 4:**
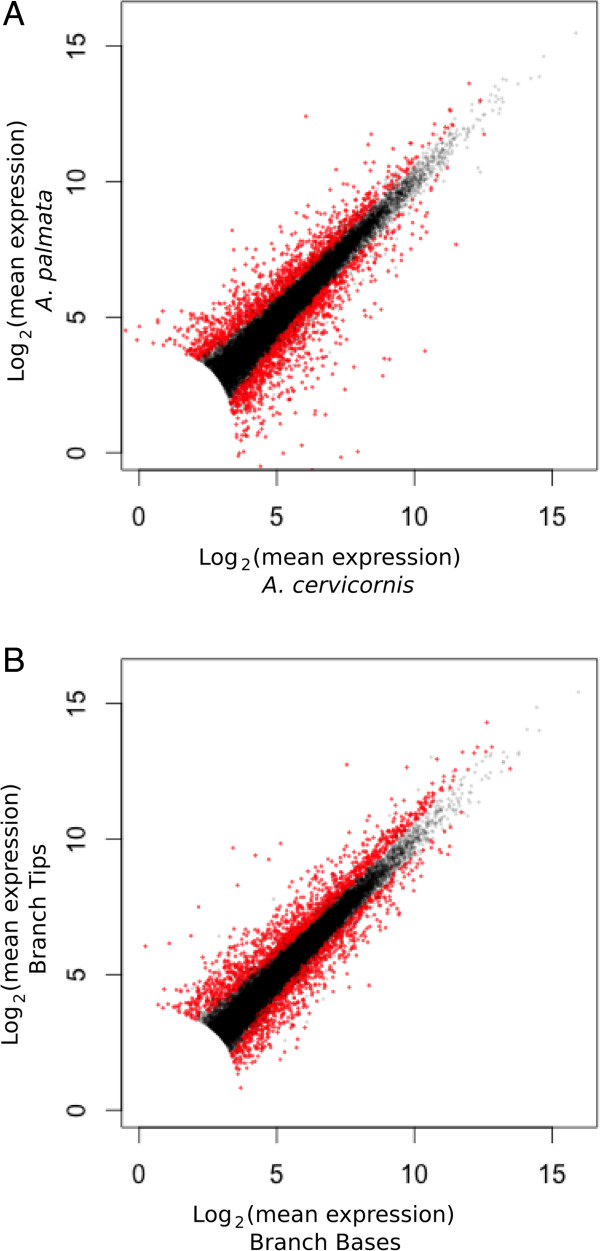
**Scatter plots of gene expression for all genes in the dataset (n = 22,320).** Species **(A)** and colony position **(B)** comparisons with DE transcripts in red (P_adj_ < 0.05). Filtering of transcripts expressed at < 100 total normalized counts results in the loss of points around the origin.

**Figure 5:**
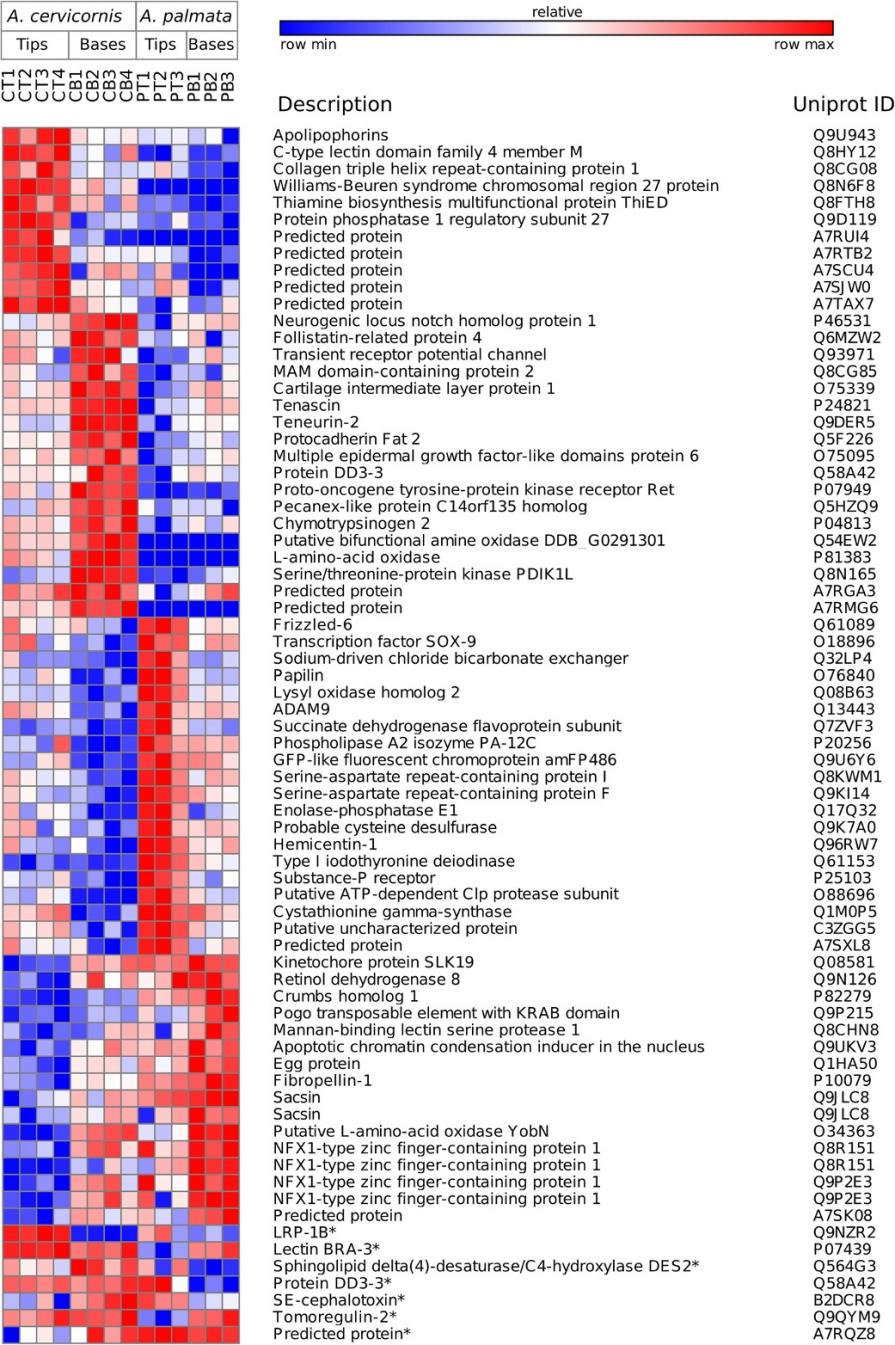
Heat map of transcripts DE for both factors or the interaction between factors (*). Includes only transcripts with fold change > 2, except for transcripts with a significant interaction effect.

A two-factor, negative binomial generalized linear model (GLM) was used to identify differentially expressed (DE) genes that differed significantly due to colony position and species or that had an interaction effect between factors (Figure 3; for annotated DE genes see Additional file 2). Out of the 2288 transcripts DE by species, 50% were up-regulated in *A. cervicornis* and 50% were up-regulated in *A. palmata* (Figure 4A). Out of the 2215 transcripts DE by colony position, 60% were up-regulated in branch tips and 40% were up-regulated in bases (Figure 4B). To elucidate the differences associated with DOL between branch base and branch tips consistent for both species, we focus on the 679 annotated transcripts DE with > 2-fold change in gene expression as a function of colony position only. To understand the genetic underpinnings of differences in growth morphology between *A. cervicornis* and *A. palmata*, we focus on transcripts that were DE for both colony position and species with > 2-fold change in gene expression or were significant for the interaction between factors. A total of 315 transcripts were DE for both factors and nine transcripts showed a significant interaction between factors. Of these, 69 transcripts were annotated with known or predicted proteins (Figure 5).

### Biological function of DE genes

Within an *Acropora* colony, branch tips are the site of rapid growth, a process including both asexual reproduction of polyps and skeleton deposition, each regulated by a number of biological functions. The basal/radial polyps of the colony are the site of gamete synthesis and energy production via the photosynthesis carried out by their higher concentration of *Symbiodinium*. Asexual reproduction of polyps requires mitotic cell proliferation as well as regulation of cell identity through developmental signaling pathways. Production of the calcium carbonate skeleton is dependent on maintaining a high aragonite saturation state at the site of calcification [51] and controlling the shape of the precipitated biomineral, most likely through extracellular matrix (ECM) proteins. As expected, genes associated with these processes were significantly DE.

**Figure 6:**
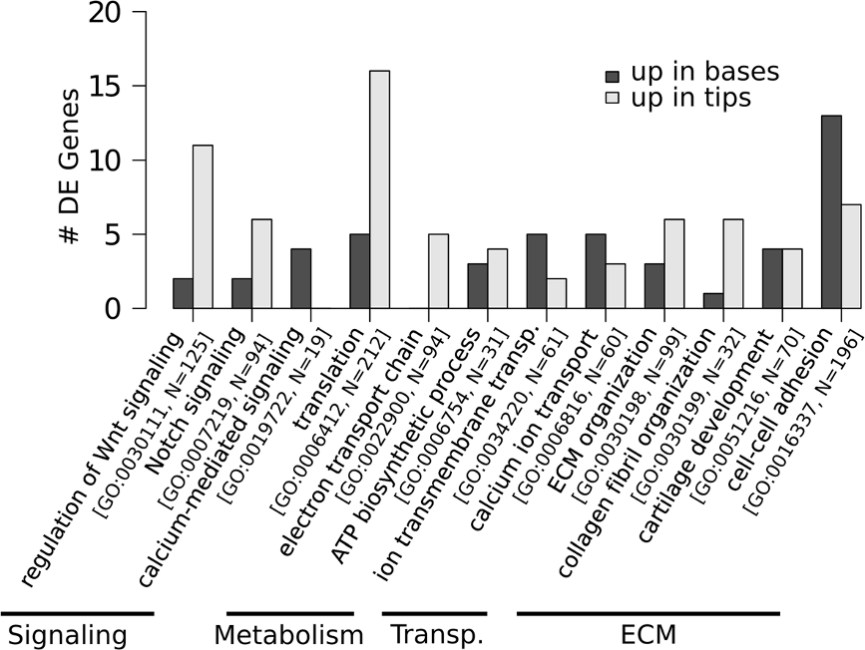
**GO term summary of DE genes for colony position.** Values represent the number of transcripts with > 2-fold change in gene expression for selected ‘enriched’ GO Biological Process terms. N is the total number of transcripts in the dataset annotated with the given GO term.

Some Gene Ontology (GO) Biological Process categories contained a relatively high number of DE genes for the colony position factor (Figure 6). Relevant categories include those involved in signaling and pattern development, metabolic processes, transport, and ECM. Categories containing many more genes up-regulated in tips include regulation of Wnt Signaling, translation, electron transport chain, ATP biosynthesis, ECM organization and collagen fibril organization. Cell-cell adhesion and calcium ion transport showed greater up-regulation in bases. To evaluate a broader number of biological functions, additional analyses were conducted based on UniProt annotation information and a review of the literature for DE genes showing greater than 2-fold difference in gene expression.

### Signaling and development

**Figure 7:**
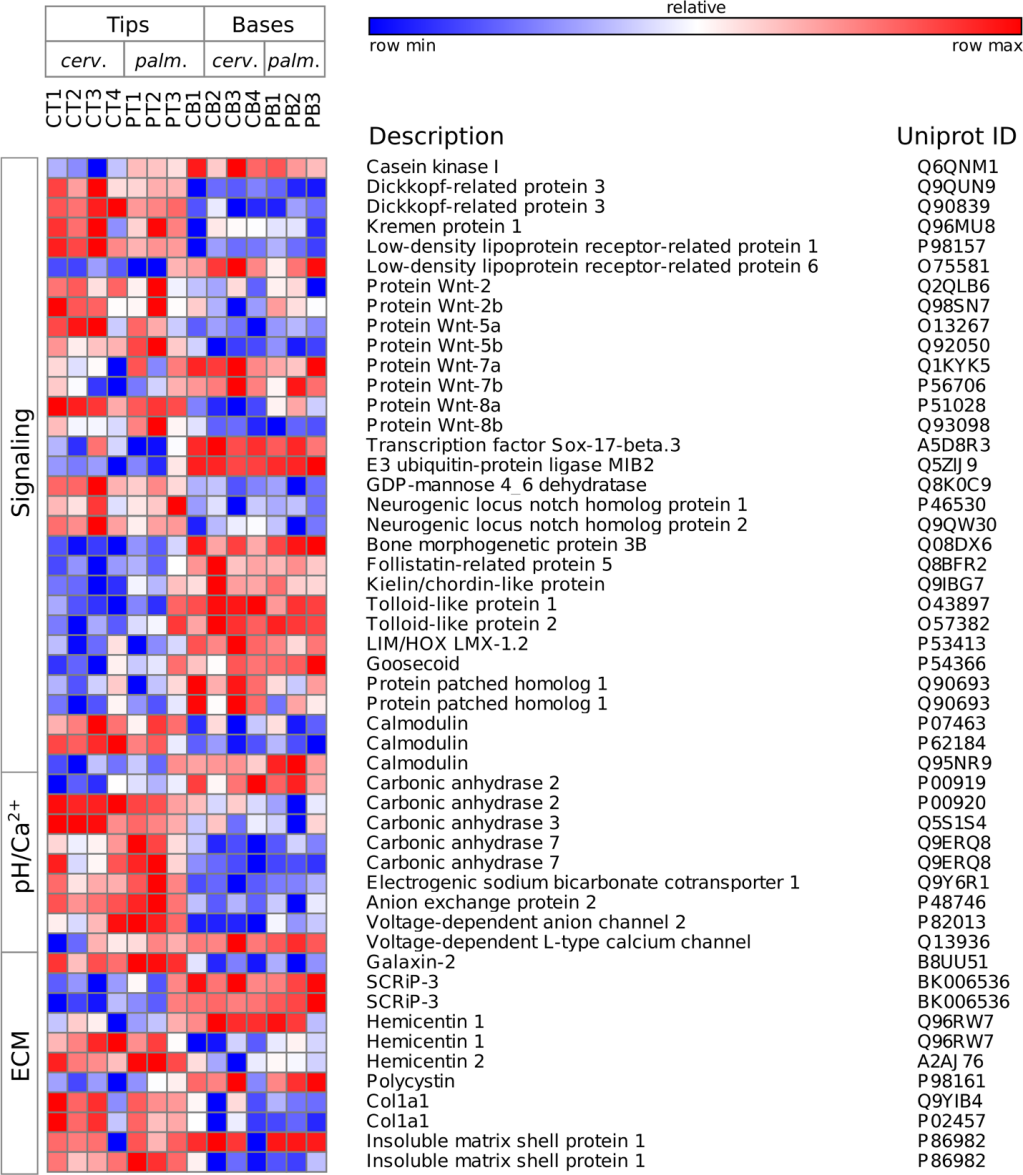
**Heat map of selected transcripts DE by colony position (but not species).** Transcripts include those putatively involved in signaling, pH regulation or ion transport, or ECM with > 2-fold DE between branch bases and tips.

There were pronounced patterns of DE signaling genes between branch tips and bases, particularly associated with three major signaling pathways, Wnt, Notch and Bone Morphogenetic Protein (BMP) (Table 1, Figure 7). These pathways are involved in pattern specification, cell fate commitment, establishment of tissue polarity, regeneration, and biomineralization and have previously been identified in cnidarians, such as *Hydra*, *N. vectensis*, and *A. millepora* [18, 52]. Differential expression of these signaling pathways within the coral colony supports their roles in reef coral development, not only during embryonic and early polyp stages, but also throughout the life of the colony.

**Table 1:** **Genes from developmental signaling pathways**

Signaling pathway	Gene family	Reference	Dataset	Pos.	Both	Int.	Trend
Wnt	Wnt proteins	13 (27)	10 (17)	8	-	-	Up tips
	Frizzled	8 (16)	8 (12)	-	1	-	Up *A. palm* tips
	Fzd-related	2 (4)	2 (4)	1	-	-	Up tips
	LRP	9 (45)	8 (25)	1	-	1	Up *A. cerv* tips
	Kremen	1	1	1	-	-	Up tips
	Dkk3	1 (6)	1 (6)	4	-	-	Up tips
	Sox9	1 (2)	1 (2)	-	1	-	Up *A. palm* tips
	Ror2	1 (3)	1 (3)	-	-	-	-
	Axin	1	1	-	-	-	-
	ß-catenin	1	1	-	-	-	-
	GSK-3ß	1 (2)	1 (2)	-	-	-	-
	Dishevelled	1	1	-	-	-	-
	Sprouty	2 (3)	2 (3)	-	-	-	-
	Wnt inhibitory factor	1 (2)	1	-	-	-	-
Notch	Notch	5 (53)	5 (23)	5	1	-	Up *A. cerv* tips
	Delta/Delta-like	6 (10)	2 (3)	-	-	-	-
	Jagged	4 (8)	3 (4)	-	-	-	-
	Hairy/enhancer of split	1 (3)	1 (3)	-	-	-	-
	E3 ubiquitin ligase MIB	2 (18)	2 (14)	1	-	-	Up bases
	Suppressor of hairless	2 (2)	2 (2)	-	-	-	-
	Numb	1	1	-	-	-	-
BMP	BMP	7 (15)	4 (10)	1	-	-	Up bases
	BMP receptor	2 (2)	2 (2)	-	-	-	-
	Chordin/chordin-like/kielin	3 (9)	3 (9)	1	-	-	Up bases

Number of genes (transcripts in parentheses) found in annotated coral transcriptome (Reference), and in the dataset. Numbers of DE genes for colony position (Pos.) and both factors (Both) indicate transcripts with > 2-fold change in expression.

In addition, we observed a small number of genes associated with these signaling pathways that were DE for both colony position and species factors. We propose that these intercellular signaling pathways, particularly Wnt signaling, may play an important role in organizing the asexual development of new polyps at branch tips and in regulating the branching patterns of *A. cervicornis* versus *A. palmata*.

### Wnt signaling pathway

Wnt signaling functions via the interaction of Wnt ligands and two types of receptor molecules, Frizzled (Fzd) receptors and low-density lipoprotein co-receptors. Wnt pathway inhibitors include Dickkopf proteins (Dkk), Wnt inhibitory factor and secreted Frizzled-Related Proteins. Studies in multiple cnidarian species, including *Hydra, N. vectensis*, *Clytia hemisphaerica* and *Hydractinia echinata,* indicate that expression of Wnt and Dkk proteins are involved in oral/aboral axis formation, head regeneration and tentacle formation [24, 27, 28, 53–55]. Wnt signaling also appears to direct axial patterning in cnidarian larva, playing a similar role to that of Hox signaling in bilaterians [12]. A number of Wnt genes DE in this dataset are either associated with developmental regulation in cnidarians or have been implicated in regulating biomineralization or cartilage formation in vertebrates (Table 2), suggesting that in calcifying corals Wnt signaling may also participate in skeleton formation.

**Table 2:** **Summary of selected DE Wnt pathway genes**

Gene	Expression higher in:	Functions & interactions	Study system	Ref.
Wnt2, 2a, NvWnt2 (AAW28132)	Tips	Oral/aboral axis determination	*N. vectensis*	[12]
Wnt5a	Tips	Oral/aboral axis determination (NvWnt5)	*N. vectensis*	[12]
		Bud/tentacle formation (hvwnt5)	*Hydra*	[56]
Wnt7a, 7b, NvWntA (AAT02182)	Bases	Oral/aboral axis determination (NvWntA; NvWnt7)	*N. vectensis*	[12]
		Bud formation/head regeneration (HyWnt7)	*Hydra*	[57]
Wnt8a, 8b, 5b, NvWnt8b (AAW28136)	Tips	Bud/tentacle formation (hvwnt8)	*Hydra*	[56]
Krem1	Tips	Interaction w/Dkk3	*Amphioxus*	[58]
			Human cells	[59]
Dkk3	Tips	Regulation of biomineralization & beak shape;	Darwin’s finches (*Geospiza* spp.)	[60]
		Migration of mature cnidocytes	*Hydra*	[61]
LRP6	Bases	Bone formation	Mouse	Review [62]
Cthrc1	A. cerv tips	Activates Wnt-PCP pathway;	Mouse	[63]
		Inhibits type 1 collagen; BMP4 & TGF-ß signaling	Mouse and rat	[64]
Apolipophorins	A. cerv tips	Inter-cellular transport of Wnt & Hedgehog signaling molecules	*Drosophila*	[65]
Fzd6	A. palm tips	Represses canonical Wnt signaling;	Human cells	[66]
		Nail/claw formation	Mouse	[67]
Sox9	A. palm tips	Cartilage development	Mouse cells	[68]
			Mouse	[69]
LRP1, 1b	Tips & interaction	Regulates Wnt pathway	Human cells	[70]

Genes include those with roles in cnidarian development and/or cartilage development and biomineralization in other organisms, as well as genes for putative Wnt-interacting proteins. Putative functions and interactions are based on the references. Closest identified cnidarian homolog and GenBank accession ID included for Wnt proteins where applicable.

Fourteen Wnt-related genes were DE between colony bases and tips for both species. The majority of these transcripts were more highly expressed in branch tips (*wnts 2, 2b, 5a, 5b, 8a & 8b, lrp1, krem1*, and *dkk3*), but five genes showed higher expression in colony bases (*wnts 7a & 7b, lrp6, sox17* and *casein kinase I*). One putative Wnt regulatory gene, *dickkopf-related protein 3* (*dkk3*), was represented by four transcripts exhibiting high up-regulation in branch tips (3.4-7.5 fold change). Although *dkk3* has shown some Wnt-related activity in bilaterians, in cnidarians there is evidence that *dkk3* facilitates migration of mature cnidocytes from the gastric region towards the tentacles [61].

Four putative Wnt pathway transcripts were significantly DE for both species and colony position, all of which were up-regulated in branch tips. Two transcripts were significantly up-regulated in A*. cervicornis* branch tips, *collagen triple helix repeat-containing protein 1* (*cthrc1*) and *apolipophorins*, and two were up-regulated in *A. palmata* branch tips, *fzd6* and *sox9*. Another gene, low-density lipoprotein receptor-related protein, *lrp1b*, which was DE by colony position and significant for the interaction between factors, is closely related to LRP1, a Wnt regulator [70]. *lrp1b* was more highly expressed in tips of both species but to a much greater degree in *A. cervicornis* (110-fold) than *A. palmata* (8-fold change).

### Notch signaling pathway

Canonical Notch signaling occurs between adjacent cells and involves a transmembrane surface receptor (Notch) that interacts with membrane-bound ligands (Delta-Serrate-LAG2) on neighboring cells [71]. Activation of Notch is associated with maintaining the undifferentiated state of cells, while suppression of Notch is required for cells to progress toward a specific cell fate [71]. In cnidarians, Notch signaling is involved in asexual budding and tentacle formation, as well as development of neural cells, oocytes and cnidocytes, as shown in developing *Hydra* [16, 72] and *N. vectensis* [17]. However, *Hydra* and *N. vectensis* are both non-calcifying cnidarians and Notch may have additional roles in calcifying corals. The effect of Notch signaling may also be influenced by interactions with Wnt and TGFß/BMP signaling pathways [73, 74].

Eighteen transcripts in the Notch signaling pathway were DE by colony position and were usually up-regulated in tips. In our dataset, *notch1* and *notch2* transmembrane protein genes and a regulatory gene, *GDP-mannose 4,6, dehydratase*, were consistently up-regulated in tips, while one Notch regulatory gene, *E3 ubiquitin-protein ligase MIB2*, was up-regulated in bases. One Notch transcript (*notch1*), which was up-regulated in *A. cervicornis* base samples, was significantly DE for both factors.

### Bone morphogenetic protein signaling pathway

BMPs are secreted signaling molecules that bind to transmembrane BMP receptors (I & II) and initiate a downstream signaling cascade regulating the expression of target genes. BMP inhibitors include chordin, noggin, and intracellular inhibitory proteins, while tolloid-like proteins may cleave chordin to enhance signaling [75]. All six BMP-associated DE genes for colony position showed higher expression in branch bases (*bmp3b*, *follistatin-related protein 5*, *kielin/chordin-like protein*, *tolloid-like protein* (*tll*) *1 & 2*, and *transcription factor scleraxis*), revealing the opposite pattern from Wnt and Notch signaling pathways. One potential BMP-related gene, *follistatin-related protein 4* (*fstl4*), which shares similarities with follistatin, a BMP inhibitor [76], was DE for both factors and was up-regulated in *A. cervicornis* bases.

BMP signaling, specifically via BMP2/4, chordin and tolloid, is important in the dorsal-ventral (D-V) axis determination of bilaterians [77, 78]. *bmp2/4* and *chordin* show localized expression during cnidarian larval development [79–81], and BMP2/4 has also been localized to the calicoblastic epithelium of mature corals, suggesting involvement in skeleton formation [15]. As its name suggests, BMP is often associated with bone growth and biomineralization, and the combined effects of BMP4 and calmodulin have been proposed to determine the overall length and width of bird beaks and fish jaws [82]. The differential expression of BMP signaling pathway (up-regulated in bases) and *calmodulin* (two transcripts up in tips, one up in bases; *see calcium signaling*) may be involved in coral biomineralization as well. Interestingly, the only BMP protein DE by colony position was *bmp3b*, which was up-regulated in bases. In vertebrate models, BMP3 and BMP3b function differently than other BMP proteins and are antagonists of osteogenic BMP2 [83–85]. Consequently, lower *bmp3b* expression in branch tips may enhance activity of other BMP proteins that were not DE by colony position, including BMP2/4.

### Other developmental signaling pathways

Hox genes encode homeodomain-containing proteins, regulatory proteins that direct patterning and identity of embryonic regions in animals. In cnidarians, some Hox/ParaHox genes appear to be involved in anterior/posterior patterning during larval development, development of sensory cells [9], and determination of polyp morphotype (*cnox2*) [32]. Most of the Hox/ParaHox genes DE by colony position, including *Hox/LIM* proteins, *goosecoid*, *paired-like*, and *six3/6* homeobox genes [86–89], have likely roles in larval or polyp development. Some transcripts annotating to Hox or Hox-like genes were DE in this dataset, yet none were DE for both factors with >2-fold change, suggesting that these genes function similarly in both *A. cervicornis* and *A. palmata*.

Two forkhead domain containing proteins and a Hedgehog pathway receptor were DE by colony position. Some forkhead box proteins are involved in embryonic development of *Hydra* [90] and *N. vectensis* [91], and many forkhead transcription factors interact with other signaling pathways, including Wnt, TGF-ß, and Hedgehog [92]. *patched homolog 1*, a Hedgehog pathway receptor associated with the divergence of jaw morpohologies in cichlid fishes [93], was up-regulated in branch bases.

### Calcium signaling

Calcium signaling relies on the gradient of calcium ions, rather than biomolecules, and is found in both prokaryotes and eukaryotes [94]. In cnidarians calcium signaling may affect multiple functions including reproduction [95], nematocyst regulation [96] and biomineralization [97]. Eight calcium signaling genes were DE by colony position. While GO term analysis indicated up-regulation of calcium signaling in bases, this is misleading because a number of calcium signaling-related genes up-regulated in tips were not annotated with this GO category, including *calmodulin*, *dysferlin* and *delta-latroinsectotoxin-Lt1a*. Both *delta-latroinsectotoxin-Lt1a*, a putative toxin [98], and its likely receptor, *latrophilin-1*, were up-regulated in branch tips. Five calcium signaling genes up-regulated in bases included a transcript of *calmodulin*, *metabotropic glutamate receptor 1*, *extracellular calcium sensing receptor*, *calcium/calmodulin dependent protein kinase*, and *E-selectin*. Calmodulin is a highly-conserved calcium-binding protein that interacts with other proteins to facilitate calcium signaling and is associated with shaping craniofacial morphology of some bird and fish species [82, 99, 100].

### Skeleton deposition

Skeletal growth in *Acropora* proceeds by a lattice-like arrangement of extending parallel ‘rods’ and reinforcing perpendicular ‘bars’ that construct the corallite [5, 101, 102]. Corallites of *A. cervicornis* are comprised of four concentric rings of skeleton [5], while those of *A. palmata* contain three concentric rings (Gladfelter, E. H., personal communication). The linear extension growth rates of these two species have been estimated at 6.5-20 cm/yr for *A. cervicornis* and 5–10 cm/yr for *A. palmata* [103–105], with *A. cervicornis* also demonstrating a faster rate of calcium deposition (μg Ca/mg N/hr) [39]. Despite the broader branches of *A. palmata* and its ability to withstand higher wave energy, skeletal construction in *A. cervicornis* is stronger and slightly less porous [106]. Our results, described below, suggest that many of the putative calcification genes are similarly expressed between species, such as carbonic anhydrase (CA), calcium ion transport proteins and ECM proteins like galaxin. Differences between species may be related to bicarbonate transport or ECM proteins that have been linked to coral skeleton or biomineralization in other organisms.

### Regulation of pH, carbonate and calcium

In scleractinian corals, mineralization of calcium carbonate occurs beneath the calicoblastic epithelium as the conversion of calcium ions and carbonate to the aragonite form of calcium carbonate (Ca^2+^ + CO_3_^2−^ → CaCO_3_) [107]. In practice the source of inorganic carbon for calcification may also be bicarbonate (Ca^2+^ + HCO_3_^−^ → CaCO_3_ + H^+^) or carbon dioxide (Ca^2+^ + CO_2_ + H_2_O → CaCO_3_ + 2H^+^), both of which produce protons that must be removed from the site of calcification [107]. Corals actively contribute to calcification through CA activity (interconversion of CO_2_ + H_2_O and HCO_3_^−^ + H^+^), and by regulating proton (i.e. pH) [108, 109] and calcium ion concentrations [109–111] within the calicoblastic epithelium and in the sub-epithelial space. Previous work has confirmed the presence and importance of CAs, anion channel, and calcium channel proteins in coral calcification and within the calcifying tissues [34, 112–114]. Though calcium ion transport across the oral epithelial layers occurs via passive diffusion in some species [115], energy-driven calcium transport across the calicoblastic epithelium is believed to involve an L-type Ca^2+^ channel protein and Ca^2+^-ATPase coupled with an anion carrier to transport calcium ions across the oral calicoblastic epithelial membrane [109, 115, 116] and a PMCA-type calcium pump [117] to transport calcium to the extracellular calcifying site. We found that CA, bicarbonate transport, and calcium ion transport transcripts were DE by colony position.

As expected for the location of rapid calcification, *CA* activity (CAs 2, 3 & 7) was highly up-regulated in branch tips, though one transcript (*CA2*) was up-regulated in bases. No *CA* transcripts were DE for both factors. In corals, it has been proposed that CAs function to provide increased inorganic substrates for both skeleton formation (bicarbonate) [113] and symbiont photosynthesis (carbon dioxide) [118], which may explain up-regulation of *CA2* in branch bases.

One inorganic carbon transport protein, *electrogenic sodium bicarbonate transporter 1* (*SLC4A4*), was up-regulated in tips, while one, *sodium-driven chloride bicarbonate exchanger* (*SLC4A10*)*,* was DE for both factors. *SLC4A10*, which was up-regulated in *A. palmata* tips, imports bicarbonate and sodium into the cell. In corals sodium-driven bicarbonate exchangers may regulate pH and supply bicarbonate for calcification [47]. A number of other ion transporters were DE by colony position. Those up-regulated in tips included anion exchange proteins, potassium channels and sodium/potassium-transporting ATPases. Those up-regulated in bases include an organic cation transporter, four solute carriers, and a cation channel.

Calcium transport may be involved directly in transporting calcium ions to the site of skeleton deposition, but may also be related to calcium signaling. A voltage-dependent L-type calcium channel protein was DE by colony position, but was up-regulated in branch bases. Three transcripts of *VWFA and cache domain containing protein* (*cachd1*), which may be involved in regulating voltage-dependent calcium channels, were DE by colony position (two transcripts were up-regulated in bases, one in tips). Transcripts of Ca^2+^-ATPase were observed in the dataset, but none was DE by colony position. Another transcription-based study of acidification found no change in the expression of Ca^2+^-ATPase in response to CO_2_-driven acidification [49]. One calcium transport gene was DE for both factors. *transient receptor potential channel*, a calcium entry channel, was up-regulated in *A. cervicornis* bases. A similar gene was previously found to be down-regulated in *A. millepora* in response to ocean acidification [49].

### Extracellular matrix (ECM) & skeletal organic matrix (SOM)

The aragonite crystals that form at the margins of growing coral skeleton resemble abiotically precipitated calcium carbonate, leading some to suggest that coral skeletal growth is independent of SOM [119, 120], yet many studies suggest a role for SOM in some form [121–127]. ECM proteins can be incorporated into the skeleton (as skeletal organic matrix; SOM) and/or provide a structured boundary for the growing skeleton. SOM is believed to control skeletal growth by either inducing or inhibiting nucleation of biomineral crystals, but some SOM proteins may be components of epithelial ECM and cell-adhesion proteins that become incorporated into the skeleton as it grows.

Until recently only one SOM protein, galaxin, had been characterized from a scleractinian coral [122], yet a number of putative SOM constituents have been proposed, including small cysteine-rich proteins (SCRiPs) [128], unidentified proteins or short peptides with high acidic amino acid (aspartic acid and glycine) content [125], glycosaminoglycans [125], lipids [129], and chitin [130, 131]. Recently, 36 SOM proteins were extracted and identified from *A. millepora* skeleton, suggesting roles for numerous functional proteins in biomineralization, including ECM-cell adhesion proteins, enzymes, acidic proteins, and a toxin [127].

### ECM: within colony differences

One galaxin, *galaxin-2,* was up-regulated in tips of both species, while another *galaxin* was not DE for either factor. Previous studies have noted that some galaxin-related genes are expressed at different stages of development [132] or respond differently to elevated CO_2_ [49], supporting distinct roles for various galaxins in calcification or other functions. Two *SCRiP-3* transcripts were also DE, but were up-regulated in bases. SCRiPs are coral-specific proteins of unknown function, but their cysteine-rich composition has been suggested as a possible mode of interaction with the SOM protein galaxin [128]. *SCRiP-3* gene expression has previously shown association with larval development [133, 134], as well as localization to developing skeletal septa [133]; however, up-regulation of *SCRiP-3* in bases suggests it is not involved in rapid calcification in branch tips of *A. cervicornis*. A number of genes DE by colony position resembled those characterized in *A. millepora*, including a *mucin*, *hemicentin*, *polycystin-1*, protocadherins and *collagen type I alpha I*. Additional ECM genes that were DE, including proteoglycan, glycoprotein and endopeptidase transcripts, may regulate cell-cell or cell-matrix interactions that guide coral growth; however, further studies are needed to determine why these genes were DE, since they may have alternate roles in corals.

One mucin, *integumentary mucin C.1*, was up-regulated in tips and may also serve as a component of the ECM or even play a role in biomineralization, as they do in molluscs [135]; however, mucins are used by corals for feeding, as a physical barrier against microbes and physical stresses [136]. Four hemicentin (1 and 2) transcripts were DE by colony position, but with up-regulation in tips and bases. In corals, hemicentin is involved in hemidesmosome-mediated attachment of the calicoblastic epithelium to the skeleton [137]. As the skeleton grows, hemicentin may become incorporated with the deposited aragonite [138]. Four cadherins or protocadherins were DE by colony position. One gene, *protocadherin Fat 3*, was up-regulated in tips, and three genes, *protocadherin Fat 4*, *protocadherin-23*, and *cadherin EGF LAG seven-pass G-type receptor 3*, were up-regulated in bases. Cadherins form adherens junctions, regulate cell adhesion, mobility and communication, but also interact with ß-catenins and are one way in which Wnt proteins may be involved in mediating cell-cell interactions [69].

Only one collagen (col1a1) has been characterized from *A. millepora* SOM, but our data indicate that a large number of additional collagens are involved in coral growth. This is not surprising given that collagens comprise about 30% of the total protein content of animals. Twelve collagen transcripts were DE by colony position, with the majority (n = 11), including *col1a1,* more highly expressed in tips. In addition, four collagen-interacting genes were up-regulated in tips, including *loxl2*, *transmembrane prolyl 4-hydroxylase* (*P4H-TM*), *collagenase 3* and *fibronectin*, and two were up-regulated in bases, *procollagen C-endopeptidase enhancer 1* and *peroxidase mit-7*.

Eleven proteoglycan and proteoglycan synthesis genes were DE by colony position. These included two transcripts of *insoluble matrix shell protein 1* (*ISMP-1*) that were up-regulated in bases and one transcript up-regulated in tips. ISMP-1 was originally identified as a component of the organic matrix in the calcified shell of the Manila clam, *Venerupis philippinarum,* and may represent a conserved biomineralization protein [139] *.* Six glycoproteins were significantly DE by colony position, including *uromodulin* (Tamm-Horsifall protein), which had two transcripts up-regulated in bases. Uromodulin controls crystal formation in the vertebrate urinary tract and may act as a mineralization inhibitor during skeleton formation, but it has also been implicated in symbiont interactions in non-calcifying cnidarians [140].

### ECM: differences between species

Three ECM proteins, similar to those found in coral SOM [127], were DE for both factors or showed an interaction between factors. *hemicentin-1*, was up-regulated in *A. palmata* branch tips. A protocadherin (*Fat 2*) transcript was most highly expressed in *A. cervicornis* bases. And a gene annotated as *cephalotoxin* from the squid *Sepia esculenta* showed an interaction between factors, though it was not significantly DE for either; it was up-regulated in branch bases of *A. cervicornis* but branch tips of *A. palmata*.

Since no collagen genes were DE for both factors, direct gene expression of collagens does not appear to determine the morphological differences between *A. cervicornis* and *A. palmata*; however, two collagen-interacting protein genes were up-regulated in branch tips as well as being DE for species. *cthrc1*, a secreted glycoprotein with putative roles in regulating deposition of extracellular collagen matrix and Wnt signaling, was up-regulated in *A. cervicornis* tips*,* and *lysyl oxidase homolog 2* (*loxl2*), which may have a role in collagen processing, was up-regulated in *A. palmata* tips.

Two C-type lectins were most highly expressed in *A. cervicornis* tips, one of these, Lectin BRA-3, is believed to be involved in biomineralization in barnacles and has been shown to either inhibit or promote crystal growth of calcium carbonate in vitro, depending on the conditions [141–143]. Lectins have numerous functional roles in animals, including cell adhesion, glycoprotein synthesis, and immunity and may regulate host-symbiont interactions in cnidarians [50, 144, 145].

### Growth & metabolism

Increased cellular activity in branch tips was indicated by high expression of genes associated with aerobic respiration and translation relative to branch bases. This is consistent with the observed higher metabolic rate at the tip of *Acropora* branches, relative to the bases [40]. However, cytoskeletal construction was up-regulated in branch bases, and genes related to ATP biosynthesis, carbohydrate and lipid metabolic processes were up-regulated both in tips and bases.

Consistent up-regulation of translation in tips of both species was indicated by a large number (n = 16) of moderately elevated (~2-fold) ribosomal protein (RP) transcripts, only two RPs were up-regulated in bases. Elevated aerobic respiration in tips was indicated by increased expression of five genes involved in the mitochondrial electron transport chain. One gene, a succinate dehydrogenase, was DE for both factors and was most highly expressed in *A. palmata* tips. Cytoskeletal construction was up-regulated in branch bases. Almost all cytoskeleton-related genes (including *actin*, *tubulin* and *dynein*) that were DE by colony position (n = 31) were up-regulated in bases (n = 27). Only four transcripts of cytoskeleton genes were up-regulated in tips (*neurofilament medium polypeptide*, *girdin*, *tubulin alpha-1D chain*, and *spectrin*).

Sphingolipid metabolism was the most prominent DE lipid biosynthesis pathway. Sphingolipids are lipids with a backbone of sphingoid bases that form a protective layer outside of the cell membrane, and complex glycosphingolipids can be involved in cell recognition, signaling, and immunity. Three genes involved in sphingolipid metabolism were DE by colony position with two up-regulated in tips (*alkaline ceramidase* and *galactosylceramide sulfotransferase*) and one up-regulated in bases (*ceramide kinase*). One transcript, *sphingolipid delta(4)-desaturase/C4-hydroxylase DES2* (*DEGS2)*, was DE by species with a significant interaction effect, showing highest expression in *A. cervicornis* bases and lowest expression in *A. palmata* bases. In cnidarians, sphingolipids appear to be involved in stability of the coral-*Symbiodinium* relationship and may determine whether heat stress results in coral bleaching [146].

Genes involved in the biosynthesis of fatty acids showed a pattern of up-regulation in bases, but lipid catabolic processes appeared to be up-regulated in tips. Three genes involved in building fatty acid chains (*fatty acid synthase*, *acetyl-CoA carboxylase 1*, and *acyl-CoA desaturase*) were up-regulated in bases and at least three genes involved in fatty acid beta-oxidation (*short-chain specific acyl-CoA dehydrogenase, hydroxyacyl-coenzyme A dehydrogenase* and *long-chain-fatty-acid-CoA ligase 5*) were up-regulated in tips. Fatty-acid molecule production in bases may serve two functions: corals may store energy produced by the higher photosynthetic activity of *Symbiodinium* as fatty-acids [147], and/or radial polyps increase production of fatty acids to meet the high lipid demand of egg development [148], which is likely to be higher during the summer spawning season. Another lipid metabolism gene, *phospholipase A2 isozyme PA-12C*, which could function in the arachidonic acid pathway, in repairing oxidized membrane lipids [149] or as a toxin in the nematocyst complex [150, 151], was DE for both factors and most highly expressed in *A. palmata* tips.

### Response to light & stress

As sessile animals, *Acropora* corals are unable to change location (unless disrupted or broken by external forces) and therefore respond to environmental stimuli and stresses physiologically. *Acropora* corals use light cues (blue light of 408–508 nm) to determine their direction of growth and to initiate axial polyp development [152], and like other corals, they may also use light to adapt their polyp behavior [153] and coordinate spawning [154, 155]. Living in relatively shallow waters, Caribbean *Acropora* are exposed to high levels of UV and heat stress, as well as oxidative stress resulting from metabolism and photosynthesis. A number of light and stress response genes were DE between tips and bases, and interestingly genes DE for both factors were consistently up-regulated in *A. palmata*. This is likely due to *A. palmata* being located in shallower water and exposed to higher levels of UV light compared to *A. cervicornis*, thus requiring a greater response of chaperone proteins and potentially photoprotective pigment (GFP-like) [156, 157].

Two photoreceptor genes that could be involved in phototropic growth, *retinol dehydrogenases* (*rdh*s) *7* and *8*, were up-regulated in tips. Rdhs convert retinol to retinal, a polyene chromophore involved in animal vision. In *A. millepora*, expression of *rdh* decreased in response to transfer into laboratory conditions, likely in response to lower light conditions [158], but increased in response to heat stress [159]. Two melatonin receptors were DE by colony position. *Melatonin receptor type 1B-B* was up-regulated in tips, and *melatonin receptor type 1A* was up-regulated in bases. Melatonin production is light dependent, with increased production at night being a primary regulator of circadian rhythm in vertebrates. Melatonin has been shown to affect expansion of oral disc in sea anemone polyps (*Actinia sp*.) [160] and may be involved in polyp behavior. In Caribbean *Acropora*, polyp behavior during the day differs between radial and axial polyps; symbiont-rich radial polyps are active throughout the day to photosynthesize, but axial polyps are only extended at night to feed. Two genes involved in photoreception, *rdh8* and *crumbs homolog 1,* were DE for both factors, and both were most highly expressed in *A. palmata* bases.

Heat shock proteins (HSPs) function during times of stress, such as at elevated temperatures, when other proteins may become denatured. One HSP, *HSP70*, was DE by colony position and was up-regulated in bases. Two transcripts of *sacsin*, a DNAJ/HSP40 protein that acts as an HSP70 co-chaperone, were DE for both factors and most highly expressed in *A. palmata* bases. While HSPs protect cells from stress-related damage and may be up-regulated in bases due to higher levels of ROS during the day resulting from photosynthesis, HSP70-related genes have also been shown to interfere with Wnt-related axial development in cnidarians [161]; therefore, lower expression of these genes in tips may actually prevent interference with proper polyp development.

Another group of proteins showing differential expression were antioxidants that may be involved in redox response. Three putative antioxidants were up-regulated in tips, including *thioredoxin domain-containing proteins* (*5 & 12*) and *selenoprotein W*. One putative stress response protein, a cyan-emitting GFP-like fluorescent chromoprotein (*amFP486*), was DE for both factors and up-regulated in *A. palmata* tips. GFP-like pigment proteins, which were also found to be up-regulated in branch tips of *A. millepora* [44], are thought to provide protection from strong UV radiation [156, 157].

### Cnidarian-specific gene expression

Known cnidarian-specific genes that exhibited differential expression were those involved in cnidocyte and nematocyst development. Nematocysts are barb-containing cnidocysts that fire in response to mechanical stress to capture zooplankton prey or as defense. These organelles are comprised of an outer wall of nematocyst outer wall antigen (NOWA) and an inner wall of minicollagen containing a barb and stored toxins [162]. A number of transcripts annotating to genes involved in nematocyst development showed increased expression in branch tips, including two *minicollagen* transcripts, two *NOWA* transcripts, and a *nematoblast-specific protein* transcript.

Coral homologs of *N. vectensis* predicted proteins with no additional known function accounted for 92 transcripts DE by colony position, nine that were DE for both factors and one that was DE for species with a significant interaction. These may be cnidarian specific genes deserving of additional investigation.

### Division of labor within the coral colony

Within the *Acropora* coral colony there is strong DOL between the actively growing branch tips and the radial polyps of branch bases. Increased expression of transcripts within our data is similarly divided between these regions, indicating that both sections of the coral branch are actively regulating different genetic processes. The high number of DE genes within colonies suggests greater differentiation among polyps in Caribbean *Acroporas* than was previously found for *A. millepora* [44]. This may be a true difference due to the contrast in colony structure between the much smaller *A. millepora* and the longer branches of Caribbean *Acropora*s, which may affect the extent to which polyps display functional differences, or it may be a function of technique (high-throughput RNA sequencing vs. microarray containing ~8700 UniGene ids). However, the DOL observed in our study between branch tips and bases appears not to be as distinct as in some hydrozoans, as the proportion of total transcripts (2,215/22,320 = 10%) DE by colony position is less than that found between functionally different siphonophore zooids (3,558/19,534 transcripts = 18%) [4].

Classical developmental signaling pathways (Wnt, Notch and BMP) were highly DE between branch tips and bases, indicating roles in the growth of mature coral colonies. In particular, up-regulation of a number of Wnt-related genes in branch tips, where new radial polyps are being produced, suggests that this signaling pathway is involved in asexual polyp budding in colonial *Acropora* corals. In other cnidarians, expression of Wnt signaling genes correlates strongly with the location of oral structures and appears to determine where tentacles and buds are produced [24, 26–28, 54, 57]. In cnidarians, Notch signaling has been studied in much less depth than Wnt signaling; however, it appears to be important during development for proper cell fate determination, neurogenesis, and for establishing tissue boundaries during the budding of new polyps [16, 17, 72]. Up-regulation of Notch signaling in branch tips may be related to any of these putative functions.

Studies of BMP signaling in cnidarians are also limited, but BMP genes show localized expression in developing embryos and appear to be involved in axis determination and gastrulation [79–81]. In mature cnidarians, *bmp2/4* is preferentially expressed in the cells that regulate skeleton deposition in corals, the calicoblastic epithelium [15]. Both Wnt and BMP pathways may affect skeleton formation, as related genes are known regulators of bone morphology and biomineralization in some vertebrate species (e.g. *LRP6*, *Fzd6*, *Dkk3*, BMP) [62, 67, 99]. Whereas Wnt and Notch signaling were up-regulated in branch tips, BMP genes were primarily up-regulated in branch bases. Interestingly, the primary BMP proteins studied in cnidarians (BMP2/4 and BMP5-8) were not DE, rather genes with putative regulatory roles were up-regulated in bases, including *bmp3b*, *chordin*, *tolloid*, *follistatin* and *scleraxis*. Additionally, calcium signaling via calmodulin, a DE calcium signaling gene known to determine morphology in vertebrates [99, 100], may influence biomineralization of the coral skeleton. Because crosstalk among signaling pathways is common [73, 74, 163, 164], it is likely they are not functioning independently.

Skeletal growth by deposition of calcium carbonate occurs more rapidly in the branch tips, where we expect genes involved in calcification to be up-regulated. Though we did not observe increased expression of Ca^2+^-ATPase, a calcium transport protein suspected to be involved in calcification, we did observe differential expression of an L-type calcium channel, which is thought to regulate calcium ion transport into the calicoblastic epithelium [115]; however it was up-regulated in bases. Up-regulation of control of pH and carbonate concentrations in growing tips was indicated by overall increased expression of CA and a bicarbonate transport protein, *SLC4A4*. Other ion transport genes, including calcium transport, were up-regulated in both branch tips and bases.

Coral skeleton formation is believed to involve ECM, both as SOM and as a boundary region. In our results, *galaxin-2*, a number of proteins with similarity to *A. millepora* SOM [127] (*mucin*, *hemicentin*, *polycystin-1*, protocadherins and *col1a1*), and ECM proteins with homologs involved in biomineralization in other species (*ISMP-1*, *chondroadherin*, *uromodulin* and collagen types I, II, XI, XXVII) were DE between tips and bases. These may be important candidate genes to investigate further as research continues into the effects of reduced oceanic pH on coral calcification.

In addition to the strong signature of DE developmental signaling genes and the numerous putative skeletal growth-related genes, we observed some differences between branch bases and tips for metabolic functions, response to environmental stimuli and stress and cnidarian-specific genes. Metabolic activity and translation were up-regulated in branch tips, supporting previous findings of increased respiration at distal regions of *Acropora* branches [40]. In this region where new tissue and polyps are being produced and mitotic rate is increased [40], it is consistent that we observed an increased signature of translation and production of mitochondrial respiratory proteins (ETC.). Interestingly, cytoskeletal genes were up-regulated in branch bases, which is unexpected but may be related to the production of gametes in this region. Some carbohydrate and lipid metabolic genes were DE by colony position; in particular, we speculate that DE lipid metabolic genes may be involved with gamete production and sphingolipid metabolism genes may be involved with regulating symbionts (see next section). Up-regulation of light and stress response genes was divided between tips and bases, though *HSP70* was consistently up-regulated in bases. The DE light response genes, including *rdh*s and melatonin receptors, may be involved in phototropic growth response, tentacle behavior and/or spawning. Cnidarian-specific genes involved in nematocyst production were consistently up-regulated in growing tips, and a majority of predicted proteins annotating to *N. vectensis*, which may represent taxonomically-restricted proteins, were also up-regulated in branch tips and may have a role in growth and calcification.

### Differences between *A. cervicornis*& *A. palmata*

The two coral species investigated, *A. cervicornis* and *A. palmata*, are sister species believed to have diverged in the Caribbean Sea approximately 3 million years ago [46]. While both species exhibit branching patterns, their morphologies are highly distinct from each other and they occupy different ecological niches. Despite these differences, these species are capable of sexual reproduction to produce F1 hybrids that display an intermediate phenotype known as *A. prolifera* [45]. RNA-seq results identified many differences in gene expression between these species that may be attributable to physiology, environment, and/or response to symbiont activity. To determine how these two species regulate the processes of growth and reproduction to achieve their distinct growth forms, we evaluated the 69 annotated transcripts that were DE both by colony position and by species, or that showed an interaction between the two factors (Figure 5).

Few of these candidate genes were associated but rather spanned a number of biological functions. Results suggest that differences in growth form between *A. cervicornis* and *A. palmata* involve Wnt, Notch, and possibly BMP signaling, regulation of bicarbonate transport by a sodium-driven chloride bicarbonate exchanger, and ECM proteins. Wnt and Notch genes DE for both factors were consistently up-regulated in tips, with three Wnt-related genes most highly expressed in *A. cervicornis* tips (*cthrc1*, *apolipophorins*, *lrp1B*) and two in *A. palmata* tips (*fzd6* and *sox9*). Cthrc, Lrp1 (a protein with similarities to Lrp1B) and Fzd6 are associated with repression of canonical Wnt signaling pathways [63, 66, 70], while apolipophorins are associated with transport of Wnt and Hedgehog molecules [65] and Sox9 is a ß-catenin-interacting transcription factor known as a master regulator of cartilage development [69]. Due to its critical role in dictating polyp growth form, Wnt regulation has been proposed as a mechanism driving morphological differences among cnidarian species [165]. Our data suggest that activation/inhibition and transport of Wnt signaling are also important in colonial corals and may play a role in maintaining dominance of a single or few axial polyps in *A. cervicornis* relative to *A. palmata*. Though one Notch receptor gene, *notch1*, was up-regulated in *A. cervicornis* tips, and one tentative BMP signaling gene, *fstl4*, was up-regulated in *A. cervicornis* bases, these pathways are not well characterized in cnidarians, so it is more difficult to speculate on their roles. Notch signaling, which is necessary for cell fate specification, neurogenesis and nematocyte differentiation in cnidarians, may be up-regulated in *A. cervicornis* tips because of the faster linear extension growth rate in this species.

In addition to developmental signaling pathways, some genes that may be more directly related to skeletal growth were DE for both factors. Although *A. cervicornis* has a slightly faster rate of linear branch extension than *A. palmata*, that growth is typically driven by a single axial polyp, with a number of smaller radial polyps developing along the side of the branch. In contrast, in *A. palmata*, multiple fused axial-type polyps lead branch extension simultaneously. One gene that may be responsible for the robust skeleton of *A. palmata* is the bicarbonate transport protein *SLC4A10*, which was up-regulated in *A. palmata* tips. Interestingly, a calcium transport gene, *calcium entry channel*, was up-regulated in *A. cervicornis* bases, but this gene may be involved in other calcium-signaling related functions such as light-response and spawning [95]. A number of ECM genes showed species-specific expression, including three genes related to known *A. millepora* SOM proteins, *hemicentin*, *protocadherin Fat 2* and a cephalotoxin (from *S. esculenta*). Other ECM genes with a potential role in morphological differences between species include collagen interacting proteins (*cthrc1* and *loxl2*), and a C-type lectin involved in biomineralization of barnacles, *lectin BRA-3*. It is not understood how these ECM proteins function in corals, but they may serve as candidates for further research.

Species-specific expression of the sphingolipid biosynthesis gene *DEGS2*, which was up-regulated in *A. cervicornis* bases and down-regulated in *A. palmata* bases, may be related the regulation of the sphingosine rheostat, a regulatory mechanism that balances signaling sphingolipids involved in cell fate and immunity, and which is suspected to facilitate coral-symbiont interactions [146]. Regulation of sphingolipids may therefore differ between species due to environmental differences that affect symbiont activity, such as temperature and light exposure. Light and heat response genes were consistently up-regulated in *A. palmata*, probably due to the greater light intensity of the reef crest environment. Two light-response genes, *rdh8* and *crumbs homolog 1*, and the HSP70 co-chaperone *sacsin* were up-regulated in *A. palmata* bases. Up-regulation of *sacsin* in *A. palmata* branch bases may be attributable to increased concentration of ROS in symbiont-rich regions of branches. A GFP-like protein up-regulated in *A. palmata* tips is also possibly involved in photoprotection of coral tissues [156, 157].

These annotated DE genes, however, do not reflect the full extent of gene expression differences between species since many genes were found to be most similar to *N. vectensis* predicted proteins, which may be cnidarian-specific, and approximately 250 transcripts DE for both factors were not annotated. Further research is needed to identify the precise location and interactions of these candidate genes and to characterize their roles in coral growth. Additionally, a number of transcripts were DE for both factors, but did not meet our criterion of a 2-fold change magnitude of expression for one or both factors. Some of these, such as transcripts of *CA2* (up in tips of *A. cervicornis*), *calmodulin* (up in tips of *A. palmata*), *chordin* (up in bases of *A. palmata*) were considered as DE for colony position, but may also be involved in differences between species.

The two species investigated are closely related enough to permit hybridization [45], but display distinct morphological characteristics and occupy different habitats: *A. palmata* is found in the high-energy reef crest and *A. cervicornis* inhabits the lower energy fore- and back-reef. As expected, we found that transcriptomes for these species exhibit large differences in gene expression, many of which are certainly due to differences in environmental factors, but some of which likely indicate differences in genetic regulation of growth form. Though studies on the role of gene expression in species evolution in corals are lacking, more is known in bilaterian systems in which the roles of developmental patterning genes such as Hox, Wnt, BMP and Hedgehog have been more widely explored. Studies in model systems indicate that changes in location, magnitude and timing of expression of functionally conserved genes, particularly during development, are responsible for morphological differences between species [166]. While colonial cnidarians differ from these other groups of organisms in that they represent a more ancestral lineage, are comprised of a coordinated group of multiple modular polyps, and display indeterminate growth, our results suggest that similar genetic signaling pathways are associated with their divergent morphologies. Furthermore, the genetic regulation of biomineralization may involve genes similar to those involved in constructing the mineralized structures in higher animals. Vertebrate species with adaptive radiations of bony and cartilaginous features, such as the beaks of finches and jaws of fish, exhibit gene expression patterns and rates of genetic evolution implicating similar genetic pathways (Wnt, BMP, calmodulin) in the development of divergent morphologies that allow adaptation to varied habitats and food sources [82, 99, 100, 167, 168]. Additional research into the gene expression of candidate genes in the hybrid *A. prolifera* and in the highly diverse Pacific *Acropora* corals can provide additional insights into the role of gene expression the evolution of their growth forms.

## Conclusions

Our RNA-seq results demonstrate that there are large differences in gene expression representing a strong DOL between polyps in the growing tips of branches compared to branch bases for both Caribbean *Acropora*. The number of transcripts differentially regulated by position within individual colonies (n = 2215) is of the same magnitude as differences between the two species (n = 2288). Genes showing differing levels of expression between branch tips and bases point to roles for classical development signaling pathways (Wnt, Notch and BMP) in branch extension and polyp development. Differential expression of CA, ion transport, ECM and putative SOM genes, indicate candidates that may be involved in the active control of skeleton growth by reef-building corals. A small number of genes were identified as DE both by colony position and species, pointing to genes that may play a role in regulating the different growth morphologies between these species.

## Methods

### Sample collection & RNA extraction

*Acropora cervicornis* and *A. palmata* samples were collected in August 2009 from Crawl Cay, Bocas del Toro, Panama. Paired apical tip (top 2 cm of branch) and base (25–30 cm from the branch tip in *A. cervicornis* and >10 cm from the branch tip in *A. palmata*) samples were collected for four colonies of *A. cervicornis* and three colonies of *A. palmata. A. cervicornis* samples were collected from colonies at least 10 m. apart at 5–6 m. depth, and *A. palmata* samples were collected from colonies 4–5 m. apart at 1–2 m. depth. Samples were flash frozen, placed in TRI Reagent (Molecular Research Center, Inc., Cincinnati, OH) and stored at −80°C. Total RNA extraction was conducted using the TRI Reagent manufacturer’s protocol with an additional 75% ethanol wash step. Total RNA quality was assessed using Agilent Bioanalyzer 2100 RNA Pico Chips, and only extractions with clear distinct 18S and 28S ribosomal RNA peaks were used (RIN 5.3-8.3).

### Illumina RNA-seq library preparation, annotation and read mapping

RNA-seq library preparation, read processing, transcriptome assembly and annotation were performed as described in Libro et al. [50]. The combined *A. cervicornis*/*A. palmata* de novo transcriptome that we used as a reference transcriptome is justified based on the less than 2% divergence in nucleotide composition between species for both mitochondrial and nuclear sequences [45]. Because galaxin and SCRiP genes may be important in coral calcification and development, we performed additional BLAST for multiple galaxin proteins (UniProt: D9IQ16, D9IQ17, D9IQ18, B8UU51, Q8I6S1, A8C9K2) and SCRiP nucleotide (GenBank: FJ842102-FJ842109, EU659816, BK006534-BK006538) sequences obtained from NCBI against queries for the full reference transcriptome. RNA-seq quantification of gene expression was conducted in CLC Genomics Workbench (CLC bio, Aarhus, Denmark) using local alignments, including non-specific mappings across multiple contigs via random assignment. Default parameters were changed by lowering the length fraction to 0.4 and increasing the similarity to 0.9 to account for potential transcriptome fragmentation resulting from the short length (36 bp) of our reads.

### Statistical analysis of differential expression

To determine whether sample groups shared similar expression profiles, clustering of samples using nMDS and the Bray-Curtis similarity index were conducted in Primer v6 [169] using coral-only count data for transcripts with greater than 100 total normalized counts. Count data were normalized by library size using the DESeq package [170] in R (R Development Core Team, 2012). Permutational multivariate analysis of variance (PERMANOVA) [171] was conducted using Bray-Curtis similarity with permutation of residuals under a reduced model in Primer v6 to test the effect of the two random independent variables, species and colony position, as well as their interaction. A significant interaction indicates that the level of expression for one factor is dependent on the level of the other factor.

DE genes were identified using a two-factor negative binomial GLM test implemented using DESeq in R. This tests the effect of two independent factors, species (i.e. *A. cervicornis* vs. *A. palmata*) and colony position (i.e. branch base vs. branch tip) on the dependent variable (gene expression); the interaction between factors was also tested. Transcripts expressed at normalized counts less than 100 (sum for all 14 samples) were excluded from the analyses to prevent bias (i.e. genes expressed at a low concentration for which a small absolute change in expression would appear to be a large fold change). Transcripts with greater standard deviation than mean within any sample group (e.g. *A. cervicornis* tips) were also excluded (after [172]). An adjusted p-value < 0.05 was used to evaluate significance [173]. We further narrowed our list of important significant DE genes by applying a threshold of 2-fold (i.e. |log_2_-fold change| > 1) difference in gene expression, except for transcripts with a significant interaction effect. “Up-regulated” indicates a higher expression in the stated treatment, e.g. up-regulated in tips indicates higher expression in tips than in bases. Heat maps of selected genes were created using log-transformed normalized count data in GENE-E [174].

### Functional genetic pathway analysis

GO terms were obtained from the GO database [175] for transcripts annotated with UniProt protein IDs (BLAST e-value < 10^−5^). Functional GO categories (for Biological Process) were further identified for DE genes using ErmineJ v. 3.0 [176], which classifies genes by GO category, identifies categories with many ‘multifunctional’ genes (which may be involved in many processes other than that specified by the current GO term), and determines if a category is ‘enriched’ for DE genes. The depth of GO annotation varied among transcripts; therefore, for functional definitions described in the results we conducted additional classification of genes based on UniProt annotations and a review of the literature.

For the DE Wnt genes, an additional BLAST (blastx) was conducted against the NCBI non-redundant protein sequence database to identify the closest cnidarian homolog to more precisely describe the putative functions of these genes.

### Availability of supporting data

The reference transcriptome sequences are available on BioProject [accession number PRJNA222758, http://www.ncbi.nlm.nih.gov/bioproject/?term=PRJNA222758]. Annotations of the coral reference transcriptome (DOI:10.6070/H4NZ85NM) and raw read count data (DOI:10.6070/H4J67DX6) are available on LabArchives.com.

## Electronic supplementary material

Additional file 1: **Average and standard error of RNA-seq library sizes by sample type.** (CSV 326 bytes)

Additional file 2: **Data set of annotated (UniProt/Swiss-Prot e-val < 10** ^**−10**^ **) coral transcripts.** Includes adjusted P and log_2_(fold change) values, and putative functions of genes discussed in the manuscript. * Indicates annotation from GenBank. (CSV 2 MB)

## Abbreviations

BMP: Bone morphogenetic protein
CA: Carbonic anhydrase
DE: Differentially expressed
DOL: Division of labor
ECM: Extra-cellular matrix
GO: Gene ontology
HSP: Heat shock protein
nMDS: Non-metric multidimensional scaling
NOWA: Nematocyst outer wall antigen
rdh: Retinol dehydrogenase
SCRiP: Small cysteine-rich proteins
SOM: Skeletal organic matrix.
